# Outcome Assessments of Patients with Posttraumatic “Ultra-Time Vascular Injuries” of the Extremities

**DOI:** 10.1038/srep17913

**Published:** 2015-12-07

**Authors:** Yi-Feng Sun, Qiong-Xuan Fang, Hong-Yan Zhan, Fan Wang, Wei Cao, Gang Zhao

**Affiliations:** 1 Traumatology Department of Shandong Provincial Qianfoshan Hospital, Shandong University, Jingshi Road 16766, Jinan, Shandong 250014, PR China; 2 Liver Disease Department of the Second Hospital of Shandong University (SHSU), Beiyuan Road 247, Jinan, Shandong 250033, PR China; 3 The Fourth Hospital of Jinan city, Jinan, Shandong, 250031, PR China

## Abstract

The management of posttraumatic vascular injury that presents after 8 h, or “ultra-time vascular injury”, is daunting, and inciting recognition of this injury is vital. We retrospectively analyzed 29 patients with ultra-time vascular injuries to determine the patients’ demographic characteristics and identify the determinants for amputation and disability. The age distribution of the high-risk population was from 18 years to 40 years, which indicated that these patients had plenty of productive life remaining. Injuries to the lower limbs (79.31%) were over four times more common than injuries to the upper limbs (17.24%), and open and blunt injuries occurred most commonly. The overall rate of limb salvage was 82.76% (24/29) and limb function is excellent in 45.83% (11/24) of the patients. The remaining patients experienced different degrees of disability in their limbs, which was determined by the anatomic location of the injury, and the presence of a combined arterial and venous injury, nerve injury, and complex soft tissue injury, as well as the occurrence of compartment syndrome. Hence, we recommend limb-salvage treatment for patients with traumatic ultra-time vascular injuries, particularly for those aged between 18 years and 40 years. Furthermore, we encourage the development of limb-salvage techniques for ultra-time vascular injuries.

A traumatic major arterial trunk injury can easily lead to limb-threatening ischemia, which can endanger the viability of the extremity and/or the patient’s life^1^. Ischemic time is an important factor that affects limb survival, and physiological and anatomical studies have shown that irreversible muscle cell damage begins after 3 h of ischemia and is nearly complete after 6 h of ischemia^2^. Therefore, it is generally accepted that vascular recanalization should be performed 6–8 h after the arterial injury has occurred^3^,^4^. However, a proportion of patients with traumatic arterial injuries present after this time, because of delays in their diagnoses and referrals to specialists. The management of these injuries presents a daunting challenge, especially in patients who have associated injuries that are severe, namely, skin and soft tissue injuries, osseous damage, and damage to the neural elements. In addition, acute complications related to extremity trauma, including soft tissue edema, hypovolemic shock, compartment syndrome, and kidney failure, increase the difficulty associated with patient management in the emergency department^5^. Sporadic reports of cases have described delayed interventions after traumatic main arterial injuries. The prognostic factors are many and varied, and there is a need to improve the guidelines for limb-salvage procedures^6^.

In this study, we conducted a retrospective analysis of 29 consecutive patients who presented to the emergency department with traumatic arterial trunk injuries of their extremities more than 8 h after the injuries had occurred. We called these injuries “ultra-time vascular injuries”. We determined the demographic characteristics of these patients and identified the determinants of amputation and disability to prompt the recognition of this injury and the provision of sound and defensible guidelines for limb salvage.

## Results

### Patients’ demographic and clinical characteristics

**Figure 1:**
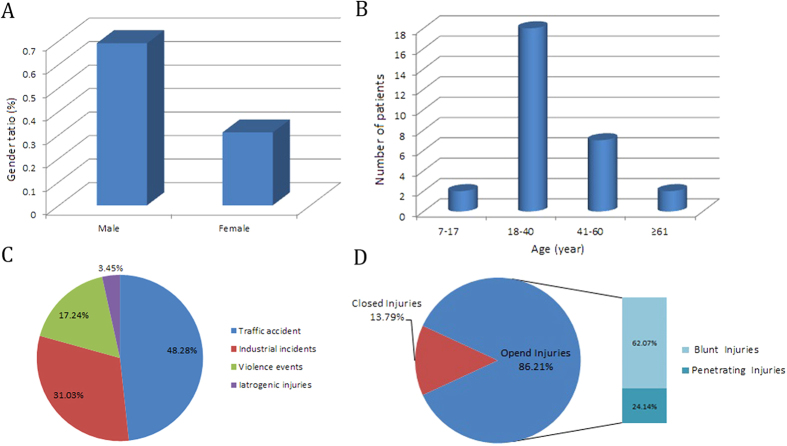
The demographics of the patients with ultra-time vascular injuries. (**A**) Male versus female incidence. (**B**) Age distribution of ultra-time vascular injuries in China. The cohort is subdivided into children and adolescents who were aged between 7 years and 17 years, young adults who were aged between 18 years and 40 years, middle-aged people who were aged between 41 years and 60 years, and older-aged people who were over 61 years of age, based on China’s age classification standard. (**C**) Vulnerability of the extremities to vascular injuries. (**D**) Contrast between open and closed mechanisms of injury, and between blunt and penetrating injuries in open trauma cases.

Twenty-nine patients, comprising 20 men and 9 women (Fig. 1A) with a mean ± standard deviation (SD) age of 33.96 ± 14.11 years, participated in this study. Fig. 1B shows that individuals who are aged between 18 years and 40 years and are defined as young adults in China^7^ are at the highest risk of ultra-time vascular injuries. The mean ± SD amount of time that elapsed between the patients sustaining the injuries and their arrival at the hospital was 11.24 ± 3.67 h, and the mean ± SD follow-up time was 18 ± 3 months. The vascular injuries to the extremities had been caused by traffic accidents in 48.28% (14/29) of the patients, industrial incidents in 31.03% (9/29) of the patients, violent events in 17.24% (5/29) of the patients, and iatrogenic injuries in 3.45% (1/29) of the patients. Of the patients, 24.14% (7/29) presented with penetrating trauma and 75.86% (22/29) presented with blunt vascular injuries. Open injuries comprised 86.21% (25/29) of the injuries, which included penetrating (24.24%) and blunt (62.07%) vascular injuries, and 13.79% (4/29) were closed injuries. The injuries occurred in the upper limbs in 17.24% (5/29) of the patients and in the lower limbs in 79.31% (23/29) of the patients (Table 1). The overall rate of amputation was 17.24% (5/29), the limb-salvage rate was 82.76% (24/29), and 45.83% (11/24) of the patients now have excellent limb functionality. Twenty-two (75.96%) patients had fractures, dislocations, or fractures combined with dislocations, 17 (58.62%) patients had complex soft tissue injuries, and 15 patients had nerve injuries. End-to-end anastomoses were possible in six (20.68%) patients, 20 (68.96%) patients required venous grafts that involved removing and interposing their saphenous veins, and three (10.34%) patients required both of these procedures.

### Determinants of amputation and disability

A multivariate logistic regression model was used to identify the determinants that were independently associated with limb amputation and disability. As shown in Table 2, the multivariate logistic regression analysis determined that the factors that were significantly associated with amputation were nerve injury (P = 0.018), complex soft tissue injury (P = 0.039), and the occurrence of compartment syndrome (P = 0.002). We then evaluated the variables and found significant differences between those patients who have excellent limb function (45.83%, 11/24) and those who experienced notable drawbacks in their limb functions (54.17%, 13/24). Multivariate logistic regression analysis determined that the variables that were significantly associated with disability included the anatomical location of the injury (P = 0.044), a combined arterial and venous (AV) injury (P = 0.047), nerve injury (P = 0.032), complex soft tissue injury (P = 0.004), and the occurrence of compartment syndrome (P = 0.009), especially the complex soft tissue injury and the occurrence of compartment syndrome contributed the most to this lack of limb function (p < 0.01). (Table 2). We also analyzed the differences between the particular anatomical sites of arterial injury in the upper and lower limbs, and found that tibial and peroneal arterial injuries more readily led to disabilities (Table 1). As a consequence, 29 patients who had complex soft tissue injuries, nerve injuries, and had experienced compartment syndrome, were more likely to lose their limbs. However, 24 limb-salvage patients who had injuries within the calf area of the lower leg, and who had complex soft tissue injuries, nerve injuries, and had experienced compartment syndrome, were more likely to lose the function of their limbs.

### Case series

Revascularization is a useful adjunct in the management of ischemic limbs after trauma. We describe three cases who presented with ultra-time vascular injuries that involved limb-threatening ischemia following trauma. These cases were successfully managed using revascularization procedures, which included end-to-end anastomoses and/or reversed saphenous vein grafting (RSVG), and good early clinical outcomes were achieved.

### Case 1

**Figure 2:**
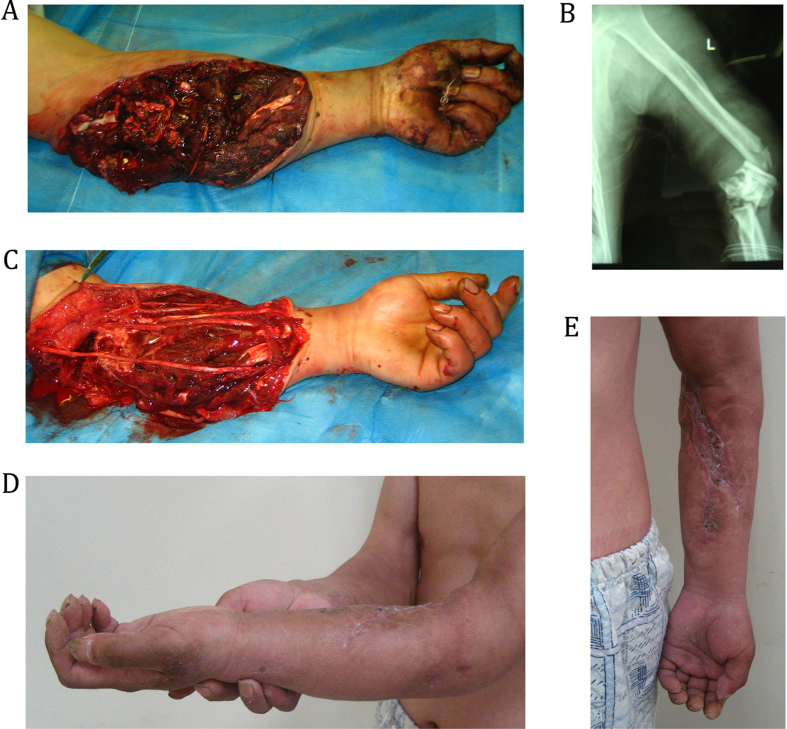
A 45-year-old man sustained a crush injury in an industrial accident and was referred to our institute after 8 h had elapsed. (**A**) Preoperative detection found that Ulnar and radial arteriovenous ruptured. (**B**) X-ray show the fracture in distal humerus and elbow joint. (**C**) End-to-end anastomoses to rebuild the vasculature. (**D,E**) The limb was successfully salvaged eventually and the patient had achieved 84 points when he was discharged.

A 45-year-old man sustained a crush injury in an industrial accident and was referred to our institute after 8 h had elapsed. He presented with bleeding, vomiting, pain, and the restriction of movement in his left forearm, and he had lost consciousness, was in shock, and he had a Type-III C open fracture (Fig. 2A,B). The left forearm was cold, clammy, pale, and swollen, and it had no pulse, had lost sensation, and the radial arterial blood flow fluctuated. The Mangled Extremity Severity Score (MESS) was 8. The intraoperative examination revealed ulnar and radial arteriovenous ruptures and accompanying nerve contusions. We created end-to-end anastomoses to rebuild the vasculature (Fig. 2C). When the vessels were recanalized, the left forearm became warm and the radial pulse was detected. The vessels were irrigated with 1% lidocaine (5 mL) and intravenous urokinase (10,000 units), and antibiotics were administered immediately. Subsequently, intermittent intravenous infusions of urokinase (100,000 units/12 h) and heparin (10,000 U/day), and injections of low molecular weight dextran and amino acids (500 mL/day) were administered for 5 days. The limb was successfully salvaged eventually. Limb function was assessed using the Fugl-Meyer upper-extremity score, and the patient had achieved 84 points when he was discharged (Fig. 2D,E).

### Case 2

**Figure 3:**
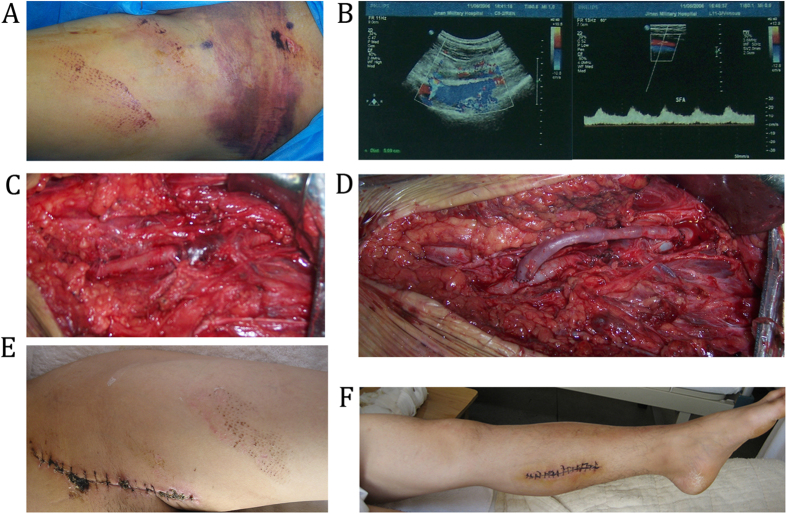
A 32-year-old man presented to the emergency department after sustaining a blunt injury to the root of his left thigh in a traffic accident. (**A**) Preoperative detection found that Blunt injury extending from the common iliac artery to the femoral artery trunk. (**B**) A color ultrasound was used to examine the vascular injury. (**C,D**) Reversed saphenous vein grafting (RSVG) to bypass the section containing the thrombus. (**E,F**) the patient recovered quickly and he was discharged from hospital after achieving a Fugl-Meyer upper-extremity score of 96 points.

A 32-year-old man presented to the emergency department after sustaining a blunt injury to the root of his left thigh in a traffic accident. Although the patient was promptly sent to hospital, no definitive diagnosis was given and the case was not given the priority it deserved. When the patient arrived at our hospital, 13 h had elapsed from the time he had sustained the injury. The initial clinical examination of the leg revealed a diffuse subcutaneous hematoma, edema, and cyanosis that were associated with the dorsalis pedis artery that had no pulse (Fig. 3A). A color ultrasound examination showed that there was a 5–6 cm thrombosis and an embolism that extended from the common iliac artery to the common femoral artery (Fig. 3B). Emergency surgery was performed using RSVG to bypass the section containing the thrombus, and the limb became ruddy (Fig. 3C,D). A preventive fasciotomy was also performed. The patient’s postoperative management and exercise regime were as described in the Methods section, and after following this postoperative management program, the patient recovered quickly and he was discharged from hospital after achieving a Fugl-Meyer upper-extremity score of 96 points (Fig. 3E,F).

### Case 3

**Figure 4:**
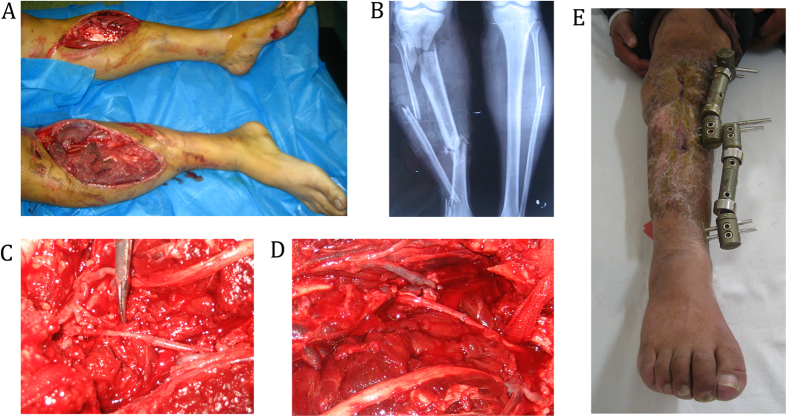
A 25-year-old man sustained open injuries to both lower extremities, with ischemia that had increased over a 10-hour period. (**A**) Preoperative detection found injuries to the anteroposterior tibial and peroneal artery. (**B**) X-ray show the fracture in tibial and peroneal.(**C,D**) The far-end peroneal artery was bridged to the popliteal artery bifurcation using RSVG, and the near-end peroneal artery was anastomosed with the distal tibial artery. (**E**) the salvage of the limb was deemed successful, and the Fugl-Meyer upper-extremity score was 89 points.

A 25-year-old man was referred to our center because he had been involved in an accident between a motorcycle and a car, and he had sustained open injuries to both lower extremities, and ischemia had increased over a 10-hour period in the right extremity. When he was admitted, the patient had hemorrhagic shock, which was indicated by his restlessness, shortness of breath, cold limbs, blood pressure of 40/65 mmHg, pulse rate of 120 beats/min, hematocrit level of 32.9%, and hemoglobin concentration of 8.7 g/dL. Correcting the shock and stabilizing the vital signs were the first steps in the patient’s treatment. During the surgical exploration, we found that the patient had sustained very serious damage to the right limb, which was classified as Gustilo IIIC, and the patient had a MESS of 8 points. Furthermore, the tibiofibular fracture was comminuted, the posterior tibial artery had contusions and thromboses that were too extensive to repair, the anterior artery had a defect that was longer than 10 cm, and the peroneal artery had not been spared and it contained a defect that was 5-cm long (Fig. 4A,B). The injuries to the left limb are not considered here. The far-end peroneal artery was bridged to the popliteal artery bifurcation using RSVG, and the near-end peroneal artery was anastomosed with the distal tibial artery (Fig. 4C,D). The limb was stabilized with a cross-knee external fixator, and, eventually, the blood supply was restored in the limb and the pulse of the dorsalis pedis artery was good. Following a postoperative management and exercise program that was tailored to the patient’s condition, the salvage of the limb was deemed successful, and the Fugl-Meyer upper-extremity score was 89 points (Fig. 4E).

## Discussion

Despite innumerable reports that describe successful limb-salvage procedures, patients who have ultra-time vascular injuries are viewed with doubt about whether salvaging the limbs is worth the risk involved, which sometimes necessitates the contemplation of limb amputation surgery rather than limb-salvage surgery^1^. Some recent publications have shown that delayed interventions can be associated with acceptable outcomes, and a small proportion of authors recommend revascularization procedures for cases that present after a long period of time has elapsed since their injuries were sustained, but delayed revascularization is associated with a higher amputation rate. The advances in clinical management have been accompanied by an increase in the number of centers employing staff who are experienced in vascular injuries, are aware of the advances in antibiotic treatments, and are knowledgeable about the usage of volume-expanding solutions and blood transfusions, which is of immense assistance to patients who are being treated for ultra-time vascular injuries^8^,^9^,^10^,^11^. In our study, the limb-salvage rate was 82.76% (24/29) and 45.83% (11/24) of the patients now have excellent limb functionality, and we are optimistic about limb salvage being worthwhile for ultra-time vascular injuries.

In China, vascular injuries account for 3% of all trauma, and vascular injuries to the extremities rank first among all vascular injuries, and they carry heavy morbidity and disability burdens^11^. The increase in the number of these injuries is probably associated with the increase in the number of motor vehicles, which has not been accompanied by the development of the infrastructure, and the mushrooming of industry that has proceeded without stringent adherence to safety precautions^12^. Of the patients who participated in this study, 48.28% had been involved in traffic accidents and 31.03% had been involved in industrial accidents. Hence, improving citizens’ traffic safety awareness and their safety awareness while they are at work are problems that require urgent solutions in China, and given that most of the patients in the current study cohort were aged between 18 years to 40 years, and raising awareness about the vulnerability of this subsection of the population to vascular injuries is critical because these individuals are able to contribute considerably to society^5^.

Ultra-time vascular injuries are complex and uncommon. Blunt vascular injuries to the extremities occurred in 75.86% of the patients in this study, and these were often accompanied by crush and avulsion injuries. Blunt vascular trauma in an extremity is an uncommon diagnosis that results in ultra-time vascular injuries^13^,^14^. In this study, the incidence of lower limb injuries was four times that of upper limb injuries. Blunt vascular injuries of the lower extremities occur most commonly in the anteroposterior tibial arteries, and repairs to this site, including fracture unions, are particularly challenging. This is associated with the characteristics of this site that include less soft tissue coverage and a higher incidence of compartment syndrome, which are often accompanied by the systemic sequelae that are associated with high-energy mechanisms^15^. Hence, guidelines regarding the monitoring of vascular statuses in blunt injuries are needed to prevent errors in judgment and to ensure the early restoration of blood flow using strict microsurgical principles. Furthermore, enabling the prompt open decompression of osteofascial compartment syndrome and ensuring proper flap plerosis should be emphasized during treatment.

There were no deaths in our patient series, but the amputation rate was 17.24%. Several factors may have contributed to this finding, including nerve injuries, complex soft tissue injuries, the performance of fasciotomies, and, in particular, artery anastomoses and graft failures. In contrast, limb-salvage procedures produced acceptable results. Starnes and Bruce reported that about 95% of injured limbs are successfully salvaged by surgical intervention and revascularization^16^. The long-term functional outcomes associated with salvaged limbs are determined mainly by the anatomic location of the injury, the presence of a combined AV injury, the presence of nerve injury, the presence of complex soft tissue injury, and whether compartment syndrome has occurred. Of the patients in this study, 75% had associated orthopedic injuries, which could be immobilized by internal or external fixation devices. While the use of internal or external fixation devices to immobilize injuries with associated orthopedic injuries is not a significant determinant of limitations in function or amputation, fractures remain an important factor if they occur in the joints. Thirteen patients (13/24, 54.17%) who underwent limb-salvage procedures had different degrees of notable drawbacks in limb function. Graham *et al.*^17^ demonstrated that major replantations had significantly better functional outcomes than prostheses. Akula *et al.*^18^ considered that lower limb reconstruction was more acceptable psychologically to patients with severe lower limb trauma compared with amputation, and that timely and regular functional training further improved patients’ lives^19^. For ultra-time vascular injuries, delayed revascularization of the limb is critical to the success of limb salvage, and it frequently involves the use of interposition grafting with autogenous saphenous vein grafts or end-to-end anastomoses^20^, and, as demonstrated by the three cases that required the establishment of individual management programs, the coordination of surgery and postoperative treatment can improve the limb preservation rate.

In the current study, the patients should have been followed up for a longer period of time to evaluate limb function, and the sample size was small. Furthermore, there does not seem to be an absolute guarantee that the sensory and motor functions of the limbs will recover, and the recoveries of these functions are determined by the anatomic location of the injury, the presence of a combined AV injury, the presence of nerve injury, the presence of complex soft tissue injury, and whether compartment syndrome has occurred. Posttraumatic ultra-time vascular injuries can be prevented and managed successfully by improving the awareness of citizens to safety issues and diseases, establishing comprehensive and individual treatment schemes, and developing sound and defensible guidelines.

## Methods

### Ethics statement

All of the patients provided written consent to participate in this study, and this enabled us to gather information from their medical records and to obtain follow-up information. This research was carried out in accordance with the principles of the Declaration of Helsinki, and it was approved by the Research Ethics Committee of Shandong Provincial Qianfoshan Hospital, Shandong University of China.

### Patient cohort and diagnosis

A retrospective analysis was conducted, and we collected information about patients who had been treated at our center between May 2011 and October 2014. We identified 29 consecutive patients who had sustained traumatic main arterial injuries to the extremities that had occurred more than 8 h before the patients presented at our institution. Patients who presented with lesions within their limbs that did not involve arterial injuries were excluded from the study. We recorded information about the patients’ demographics, the mechanisms underlying the injuries, the patients’ physiologic statuses, the length of time that the patients had sustained the injuries before receiving treatment, any associated injuries, the tests and procedures performed, the vascular operative findings, treatments received, complications experienced, and the outcomes. Compound fractures were graded according to the classification system developed by Gustilo, and mangled extremities were assessed using the MESS^21^,^22^.

The diagnoses of the vascular injuries to the extremities were made based on the clinical signs and symptoms that included pulsatile external bleeding, the presence of rapidly expanding hematomas, the absence of distal pulses, bruits over the arteries, pallor, the presence of cold distal extremities, or the presence of ischemic limbs, and these were confirmed using Doppler ultrasound or computed tomography angiography (CTA). All of the diagnostic tests were interpreted by two senior surgeons and/or radiologists.

### Perioperative treatment and surgical techniques

All of the patients underwent full physical examinations and resuscitation according to the principles of the advanced trauma and life support guidelines. When the patients had stabilized, and once the vascular injuries had been evaluated and diagnosed based on the clinical symptoms and the Doppler or CTA assessments, the patients were immediately taken to the operating room for revascularization. The patients underwent vascular repairs that included the creation of end-to-end anastomoses and/or RSVG. In some patients with polytrauma who had open injuries and/or fractures and dislocations, all of the grossly nonviable tissue and foreign bodies were thoroughly debrided and removed. The vascular repairs followed the stabilization of the fractures, because initial skeletal stabilization was necessary to prevent repeated injuries to the vessels caused by skeletal manipulations. Accompanying nerve injuries were repaired at the time of the vascular repairs, and major associated venous injuries were repaired whenever possible. Appropriate covers for the defects were developed by the plastic surgeons who used split-skin grafts or applied flap techniques.

### Postoperative management and exercise

To prevent vascular complications, including blood hypercoagulability, vasospasms, and vascular embolisms, postoperative antithrombosis therapy, preventing reperfusion damage, and fasciotomy management are critical. Hence, during the creation of the anastomoses, the arterial lumina were topically irrigated with 1% lidocaine (5 mL) and intravenous flushes of urokinase (50,000–300,000 units). The patients were then administered intravenous infusions of urokinase (100,000 U/12 h) and heparin (10,000–20,000 U/day), and they were administered injections containing low molecular weight dextran and amino acids (500 mL/day) for 5–7 days^23^. All patients received intravenous preoperative prophylactic antibiotics, which were continued postoperatively for 2–7 days. In addition, timely functional training was very important. Periodic postoperative reviews were carried out in the outpatient department to assess the functional statuses of the limbs. These assessments were undertaken once every 2 months for 6 months, then once every 6 months for a total of 2 years.

### Statistical analysis

The continuous data were compared using Student’s t test for independent samples, and the categorical data were analyzed using the chi-square test or Fisher’s exact test, as appropriate. Limb function was evaluated using the Fugl-Meyer Assessment Scale. We defined a Fugl-Meyer score >85 points as excellent and good limb function, and patients with Fugl-Meyer scores of <85 points were identified as having notable functional drawbacks^24^,^25^. Multivariate logistic regression analysis was used to examine the independent associations between different demographic and injury-related factors and the binary categorical outcome measure for limb amputation, namely, yes or no. All of the statistical analyses were performed using the IBM^®^ SPSS^®^ Statistics version 19 (IBM Corporation, Armonk, NY, USA) for. A value of P < 0.05 was considered statistically significant^26^.

## Additional Information

**How to cite this article**: Sun, Y.-F. *et al.* Outcome Assessments of Patients with Posttraumatic "Ultra-Time Vascular Injuries" of the Extremities. *Sci. Rep.* **5**, 17913; doi: 10.1038/srep17913 (2015).

## Figures and Tables

**Table 1:** Frequencies and Multivariate regression analysis of vascular injuries to the extremities according to the anatomic location.

Anatomicalsite	Artery	%	(No.)	Amputation	Disability
Arm	–	17.24%	5	0.858	0.044
	Axillary	6.90%	2	0.504	0.174
	Brachial	3.44%	1	0.642	0.347
	Radial and ulnar	6.90%	2	0.204	0.347
Lower limb	–		24	0.858	0.044
	Iliac artery	6.90%	2	0.504	0.108
	Femoral	20.69%	6	0.967	0.085
	Popliteal	13.80%	4	0.326	0.200
	Tibia	6.90%	3	0.404	0.439
	Tibial and peroneal	34.48%	9	0.124	0.009

**Table 2:** Multivariate logistic regression analysis of the determinants associated with amputation and disability.

Determinants	Amputation	Disability
Age, years	0.114	0.391
Male vs female	0.558	0.562
Upper VS Lower Limb Injuries	0.858	0.044
Combined AV injury	0.187	0.047
Associated fracture	0.166	0.476
Associated nerve injury	0.018	0.032
Associated complex soft tissue injury	0.039	0.004
Compartment syndrome	0.002	0.009
Penetrating vs blunt mechanism	0.116	0.106

## References

[b1] Langer V. Management of Major Limb Injuries. The Scientific World Journal 2014 (2014), 10.1155/2014/640430. PMC3913364 24511296

[b2] Blaisdell F. W. The pathophysiology of skeletal muscle ischemia and the reperfusion syndrome: a review. Vascular 10, 620–630 (2002). 10.1177/096721090201000620 12453699

[b3] Yilmaz A. T. *et al.* Missed arterial injuries in military patients. The American journal of surgery 173, 110–114 (1997). 9074374 10.1016/S0002-9610(96)00423-0

[b4] Padberg F. T. *et al.* Infrapopliteal arterial injury: prompt revascularization affords optimal limb salvage. Journal of vascular surgery 16, 877–886 (1992). 1460714 10.1067/mva.1992.42019

[b5] Sabapathy S. R. Management of complex tissue injuries and replantation across the world. Injury 37, 1057–1060 (2006). 17049349 10.1016/j.injury.2006.07.026

[b6] Mullenix P. S. *et al.* Limb salvage and outcomes among patients with traumatic popliteal vascular injury: an analysis of the National Trauma Data Bank. Journal of vascular surgery 44, 94–100 (2006). 16828431 10.1016/j.jvs.2006.02.052

[b7] Adamchak D. J. The effects of age structure on the labor force and retirement in China. The Social Science Journal 38, 1–11 (2001).

[b8] Moini M., Takyar M. A. & Rasouli M. R. Revascularisation later than 24h after popliteal artery trauma: is it worthwhile? Injury 38, 1098–1101 (2007). 17697677 10.1016/j.injury.2007.05.001

[b9] Zhong-Wei C., Meyer V., Kleinert H. & Beasley R. Present indications and contraindications for replantation as reflected by long-term functional results. The Orthopedic clinics of North America 12, 849–870 (1981). 7322515

[b10] Bondurant F. J., Cotler H. B., Buckle R., Miller-Crotchett P. & Browner B. D. The medical and economic impact of severely injured lower extremities. Journal of Trauma and Acute Care Surgery 28, 1270–1273 (1988). 10.1097/00005373-198808000-00023 3137367

[b11] Zhang X., Yang Q. & Shang J. Diagnosis and Treatment of Acute Vascular Injuries in Limbs [J]. CHINESE JOURNAL OF ORTHOPAEDICS 11, 008 (1999).

[b12] Wang A., Sun H. & Du Q. Diagnosis and treatment of major vascular injuries in extremities in 177 cases. Chinese Journal of Traumatology 10, 003 (2001).

[b13] Rozycki G. S., Tremblay L. N., Feliciano D. V. & McClelland W. B. Blunt vascular trauma in the extremity: diagnosis, management, and outcome. Journal of Trauma and Acute Care Surgery 55, 814–824 (2003). 10.1097/01.TA.0000087807.44105.AE 14608150

[b14] Ben-Menachem Y. Vascular injuries of the extremities: hazards of unnecessary delays in diagnosis. Orthopedics 9, 333–338 (1986). 3960771 10.3928/0147-7447-19860301-05

[b15] Mcnutt R. *et al.* Blunt tibial artery trauma: predicting the irretrievable extremity. Journal of Trauma and Acute Care Surgery 29, 1624–1627 (1989). 2593189

[b16] Starnes B. W. & Bruce J. M. Popliteal artery trauma in a forward deployed Mobile Army Surgical Hospital: lessons learned from the war in Kosovo. Journal of Trauma and Acute Care Surgery 48, 1144–1147 (2000). 10.1097/00005373-200006000-00022 10866263

[b17] Graham B., Adkins P., Tsai T.-M., Firrell J. & Breidenvach W. C. Major replantation versus revision amputation and prosthetic fitting in the upper extremity: a late functional outcomes study. The Journal of hand surgery 23, 783–791 (1998). 9763250 10.1016/s0363-5023(98)80151-2

[b18] Akula M., Gella S., Shaw C., McShane P. & Mohsen A. A meta-analysis of amputation versus limb salvage in mangled lower limb injuries—the patient perspective. Injury 42, 1194–1197 (2011). 20598306 10.1016/j.injury.2010.05.003

[b19] Whinnery S. B. & Whinnery K. W. Effects of functional mobility skills training for adults with severe multiple disabilities. Education and Training in Autism and Developmental Disabilities 46, 436–453 (2011).

[b20] Jagdish K. *et al.* The Outcomes of Salvage Surgery for Vascular Injury in The Extremities: A Special Consideration For Delayed Revascularization. Malaysian orthopaedic journal 8, 14 (2014). 25279079 10.5704/MOJ.1403.012 PMC4093557

[b21] Smith R. The classification of fractures. J Bone Joint Surg Br 82, 625–626 (2000). 10963153 10.1302/0301-620x.82b5.11129

[b22] Rush R. M. Jr *et al.* Application of the Mangled Extremity Severity Score in a combat setting. Military medicine 172, 777–781 (2007). 17691695 10.7205/milmed.172.7.777

[b23] Bueno R. A. *et al.* Replantation: Current Concepts and Outcomes. Clinics in plastic surgery 41, 385–395 (2014). 24996460 10.1016/j.cps.2014.03.010

[b24] Elmqvist L. *et al.* Knee extensor muscle function before and after reconstruction of anterior cruciate ligament tear. Scandinavian journal of rehabilitation medicine 21, 131–139 (1988). 2799311

[b25] Woodbury M. L. *et al.* Dimensionality and construct validity of the Fugl-Meyer Assessment of the upper extremity. Archives of physical medicine and rehabilitation 88, 715–723 (2007). 17532892 10.1016/j.apmr.2007.02.036

[b26] Sun Y. *et al.* Gli1 inhibition suppressed cell growth and cell cycle progression and induced apoptosis as well as autophagy depending on ERK1/2 activity in human chondrosarcoma cells. Cell death & disease 5, e979 (2014). 24384722 10.1038/cddis.2013.497 PMC4040663

